# Evaluating the Correlation Between Optical Coherence Tomography Angiography-Derived Parameters and Serum Vascular Endothelial Growth Factor-A (VEGF-A) Levels in Central Serous Chorioretinopathy

**DOI:** 10.7759/cureus.108065

**Published:** 2026-04-30

**Authors:** Punita K Sodhi, Hitenkumar Miyani Parvinbhai, Apoorva Goyal, Kirti Singh, Parul Jain, Bhawna Mahajan, Richa Asthana, Alka Yadav, Alok Kumar

**Affiliations:** 1 Ophthalmology, Guru Nanak Eye Centre and Maulana Azad Medical College, New Delhi, IND; 2 Biochemistry, Govind Ballabh Pant Hospital, New Delhi, IND

**Keywords:** central serous chorioretinopathy, cnvm (choroidal neovascular membrane), optical coherence tomography angiography, optical coherence tomography (oct), vascular endothelial growth factor a levels

## Abstract

Background and objective: Release of vascular endothelial growth factor-A (VEGF-A) is one of the mechanisms involved in the pathogenesis of central serous chorioretinopathy (CSCR). We intended to evaluate the correlation between optical coherence tomography angiography (OCTA) parameters and serum VEGF-A levels in CSCR.

Methodology: Our prospective interventional study included 31 patients diagnosed with CSCR. The subjects had a mean age of 39.23±6.9 (26-53) years. They were examined for visual acuity (VA) and tested for color perception and contrast discrimination. OCT was conducted to examine central macular thickness (CMT) and subfoveal choroidal thickness (SFCT), and to detect neurosensory detachment (NSD), pigment epithelial detachment (PED), and other abnormalities. The OCTA measured foveal avascular zone (FAZ) and vessel density (VD) across various retino-choroidal layers. The serum VEGF-A levels were measured using commercially available enzyme-linked immunosorbent assay (ELISA) kits. The eyes were treated with a single intravitreal injection of 0.3 mg/0.05 ml ranibizumab biosimilar (RbB). After one month, the ocular parameters were rechecked, and serum VEGF-A levels were remeasured. We assessed the correlation between ocular parameters and VEGF-A levels in serum samples at both instances.

Results: At baseline, the mean value of the best corrected visual acuity (BCVA) in logarithm of the minimum angle of resolution (LogMAR) was 0.81±0.43. The CMT measured 286.81±117.67 microns, SFCT 194.32±62.76 microns, and the serum VEGF-A level was 351.64 (IQR 243.57-478.71) pg/ml. Before treatment, circulating VEGF-A levels correlated positively with the LogMAR value of BCVA (Rho=0.121), CMT (Rho=0.282), SFCT (Rho=0.006), NSD height (Rho=0.100) and width (Rho=0.019), and PED number (Rho=0.016), height (Rho=0.246) and width (Rho=0.066). The serum VEGF-A showed a weak positive correlation (p>0.05) with most OCTA features, whereas it correlated negatively with FAZ area (Rho=-0.136) and perimeter (Rho=-0.128). A month after intravitreal injection, LogMAR BCVA improved to 0.42±0.42, CMT measured 256.65±109.57 microns, and SFCT was 209.84±41.53 microns. The median serum VEGF-A level was 336.31 (IQR 259.455-500.105) pg/ml. Serum VEGF-A showed weak positive correlation with LogMAR value of BCVA (Rho=0.046), CMT (Rho=0.063), SFCT (Rho=0.094), NSD number (Rho=0.290), height (Rho=0.269) and width (Rho=0.289), and PED number (Rho=0.306), height (Rho=0.204) and width (Rho=0.124). The serum VEGF-A showed negative weak correlations (p>0.05) with OCTA features, including FAZ metrics --area (Rho=-0.156), perimeter (Rho=-0.114), and circularity (Rho=-0.335), as well as VD in various retino-choroidal layers: superficial capillary plexus (Rho=-0.282); deep capillary plexus (Rho=-0.225), outer retinal chorio-capillaries (Rho=-0.295), chorio-capillaries (Rho=-0.312) and choroid (Rho=-0.274).

Conclusion: Higher serum VEGF-A was associated with worse VA and increased CMT, SFCT, NSD height and width, and PED number, height, and width. An increase in VEGF-A was related to reduced FAZ area and perimeter. At baseline, higher levels of VEGF-A were accompanied by an increase in VD in all layers except the outer retina and choroid. After treatment with intravitreal RbB, a similar pattern was observed; however, increased VEGF-A was found to be coupled with decreased VD in all retino-choroidal layers, except the outer retina.

## Introduction

Central serous chorioretinopathy (CSCR) is marked by the accumulation of serous subretinal fluid (SRF) and neurosensory retinal detachment due to hyperpermeability of choroidal vessels. It is now recognized as part of the pachychoroid spectrum disease [1]. Various factors linked to the onset of CSCR include type A personality, steroid use, hypertension, *Helicobacter pylori *infection, sleep disturbances, autoimmune diseases, and psychopharmacologic medications. The SRF accumulation in CSCR can result from breakdown of the outer blood-retinal barrier (BRB), retinal pigment epithelium (RPE) dysfunction, increased permeability of choroidal vessels, and the release of cytokines, including vascular endothelial growth factor (VEGF), placental growth factor (PGF), and interleukins [2,3].

VEGF is produced both in retinal and choroidal cells in ischemic conditions caused by diminished perfusion. This factor increases vascular permeability, leading to edema [4]. Evidence suggests that anti-VEGF therapy mitigates choroidal leakage by directly inhibiting VEGF-A, enhancing Na⁺/K⁺-ATPase expression, and reducing intracellular osmotic pressure [5]. Recent studies have shown that intravitreal injections of bevacizumab in CSCR patients lead to improvements in vision and reductions in neurosensory detachment (NSD), without any reported adverse events [6]. Furthermore, as it is a smaller molecule with greater binding affinity for VEGF, ranibizumab has better retinal penetration than bevacizumab and thus may be more effective. Clinical studies on their use in CSCR have yielded variable results [2,4,5,7,8].

Optical coherence tomography angiography (OCTA) is a novel non-invasive imaging technique that scans the retinal and choroidal vasculature by capturing the motion contrast generated by blood flow. It reveals changes in the flow patterns of the chorio-capillaries (CC), providing greater sensitivity and specificity for diagnosing choroidal neovascularisation (CNV) in CSCR than traditional angiographic methods [9]. With OCTA, vascular abnormalities such as NV can be detected at the CC level, displaying various morphologic patterns of CNV [5]. The NSD and pigment epithelial detachment (PED) can reduce retinal vascularity through pressure effect and appear as dark areas and dark spots in OCTA scans [10].

The VEGF-A is the prototypical member of the VEGF family and has been most widely studied among the various VEGFs. However, it has been rarely studied in the CSCR, and its effect on ophthalmic features and OCTA parameters remains unknown. Given the proposed role of inflammation in CSCR, we hypothesized that serum VEGF-A levels would be significantly correlated with OCTA parameters. The primary objective of this study was to assess changes in OCTA parameters in CSCR following intravitreal ranibizumab biosimilar (RbB). Secondary objectives included measuring serum VEGF-A levels before and after treatment and evaluating their correlation with clinical, OCT, and OCTA parameters at both time points.

## Materials and methods

Treatment naïve subjects, having non-resolving CSCR with the presence of NSD involving the fovea on optical coherence tomography (OCT), lasting for more than four months after the onset of symptoms, and an active angiographic leakage on fundus fluorescein angiography (FFA) were enrolled in a prospective interventional study from September 2023 to September 2024 [11]. The study took place in Guru Nanak Eye Centre and Maulana Azad Medical College. The study protocol was approved by the ethical committee of the Maulana Azad Medical College, New Delhi, vide letter number F.1/IEC/MAMC/96/02/2023/No. 225 dated 15/05/2023, and written consent was obtained from the participants. The study was conducted in accordance with the Declaration of Helsinki.

Subjects with best corrected visual acuity (BCVA) of logarithm of the minimum angle of resolution (LogMAR) value of 1.0 or better were included in the study. Patients with ocular conditions that cause SRF accumulation, such as diabetic retinopathy, retinal vascular occlusion, ocular inflammation, or conditions that cause macular exudation, such as pathologic myopia and angioid streaks, were excluded from the study. Those who had undergone any intraocular surgery other than uncomplicated cataract extraction or had a risk of thrombo-embolic events were not enrolled.

The patients were questioned regarding the onset and duration of disease, and symptoms such as blurred vision, central or paracentral scotoma, metamorphopsia, decreased contrast sensitivity (CS), or defective colour vision (CV). We took the patients' history to elicit risk factors, including steroid intake, hypertension, sleep disturbance, autoimmune disease, and psychopharmacologic medications. Visual acuity (VA) was measured using self-illuminated Early Treatment Diabetic Retinopathy Study (ETDRS) charts at 4 m under standardized lighting conditions and recorded in logarithm of the minimum angle of resolution (LogMAR) units. Refraction was performed to obtain an appropriate prescription to achieve the BCVA. The intraocular pressure (IOP) was recorded using Goldman applanation tonometry (AT 020, Carl Zeiss Meditec AG, Jena, Germany) [12]. The colour perception was tested using Ishihara plates (38th edition, 2012) at a distance of approximately 70 centimetres [12,13]. Additionally, in the Farnsworth D 15 test, the subjects arranged 15 movable-coloured discs in a specific hue sequence, beginning from a fixed reference cap at approximately 50 centimetres [12,14]. The CS was assessed using a uniformly illuminated Pelli-Robson chart (Haag-Streit Service, Inc., Ohio 45040, USA) at one meter [12]. Each affected eye was tested separately for colour sensitivity and contrast discrimination, under optimal light conditions, with subjects wearing proper refractive correction. We examined the eyes with a slit lamp for signs of anterior segment inflammation. The Amsler grid was used to examine metamorphopsia and scotoma, with the grid held at a near distance of about 33 cm. The subjects wore their reading glasses to trace the lines with a pen while focusing on the dot in the centre. Each eye was tested separately, with the other eye remaining covered. The wavy lines or differences in square size were reported as metamorphopsia, while missing lines were reported as scotoma [15].

We performed a dilated fundus examination using both direct and indirect ophthalmoscopy to evaluate the cup-to-disc ratio and detect abnormalities, including hemorrhages, RPE changes, and scars. We obtained color fundus photographs using a fundus camera (FF450plus, Visucam PRO NM; Carl Zeiss Meditec AG, 07740 Jena, Germany) [12]. The FFA was performed following the intravenous injection of 3 ml of sodium fluorescein dye to identify areas of hyperfluorescence, leakage, and ill-defined hyperfluorescent patterns suggestive of CNV. Indocyanine green angiography (ICGA) was performed to assess choroidal lobular ischemia, choroidal venous congestion, and multiple areas of choroidal vascular hyperpermeability.

A high-definition 3 mm × 3 mm OCT scan (spectral-domain OCT, RTVue; Optovue Inc., Fremont, CA) centered on the fovea was performed to measure central macular thickness (CMT), subfoveal choroidal thickness (SFCT), height and width of NSD, height and width of pigment epithelial detachment (PED), and to look for RPE irregularities, integrity of photoreceptor inner segment/outer segments (IS/OS) line and choroidal neovascular membrane (CNVM) [12]. The CMT was automatically measured as the distance between the internal limiting membrane (ILM) and the inner border of the RPE within the central subfield of the ETDRS grid. The NSD height was defined as the maximum vertical distance between the outer border of the detached neurosensory retina and the inner border of the RPE. The width was measured as the horizontal distance between the two margins of the detached neurosensory retina. The PED height was measured as the distance between the apex of the elevated RPE and Bruch’s membrane on the OCT B-scan, while the width was defined as the horizontal distance between the two edges of the detached RPE.

We performed a 3 × 3 mm OCTA scan (SD-OCT RTVue; Optovue Inc., Fremont, CA) centered on the fovea to assess the foveal avascular zone (FAZ) and retinal vasculature, including the superficial and deep capillary plexuses (SCP and DCP), outer retina (OR), outer retinal chorio-capillaries (ORCC), chorio-capillaries (CC), and choroid (Ch) [12]. The CNVM was seen as a hyperreflective, tangled vascular network in the CC layer beneath the SRF [5]. The FAZ area, perimeter, and circularity were measured. With the ETDRS grid placed at the posterior pole, the fovea was defined as the region within the central 0.5-mm ring; the parafovea as the area between the 0.5-mm and 1.5-mm rings; and the perifovea as the area between the 1.5-mm and 3-mm rings. The device acquired two OCT volumes at a scanning speed of 85,000 A-scans per second, with each volume comprising 304 × 304 A-scans, in approximately three seconds. The OCTA scans were generated using the split-spectrum amplitude-decorrelation angiography (SS-ADA) algorithm.

The OCTA software automatically segmented the six layers of retina and choroid and generated vessel density (VD) measurements for each. The SCP extended from the internal limiting membrane (ILM) to 13 μm below the inner plexiform layer/inner nuclear layer (IPL/INL). The DCP was segmented between IPL/INL and outer plexiform layer/outer nuclear layer (OPL/ONL), starting at 8 μm below the IPL/INL and extending to 13 μm below the OPL/ONL. The OR spanned from the OPL/ONL to the RPE/Bruch’s membrane (BM), covering a depth starting at 8 microns below OPL/ONL till 71 microns above RPE/BM. The ORCC was defined between OPL/ONL and RPE/BM, extending at 8 μm below the OPL/ONL to 21 μm below the RPE/BM. The CC layer extended from 4 microns to 32 microns beneath the RPE/BM, while the Ch was segmented from 25 microns to 63 microns below the RPE/BM. We calculated the VD as the ratio of the vessel area (flowing blood) to the total segmented region area expressed as a percentage.

Two independent observers (PKS and MHP) used a standardised scanning protocol to capture the OCT and OCTA images of the affected eye under consistent mesopic light conditions. The readings they recorded were averaged. We ensured that all images had a signal strength index of 90% and a signal quality index of 4/5. We used the “Auto All” function to optimise image quality. Scans with poor quality due to blinking, media opacities, motion artefacts, and projection artefacts were excluded and repeated until acceptable image quality was achieved. In cases of segmentation errors in automatically acquired scans, images were reacquired manually to improve accuracy and overall image quality.

Following withdrawal of 3 mL of venous blood by venipuncture, samples were collected in tubes containing ethylene diamine tetraacetic acid (EDTA) and allowed to stand at room temperature for 30 minutes for clot formation. The samples were then centrifuged at 3000 revolutions per minute for 15 minutes to separate the clot. The serum was carefully aliquoted and stored at −80°C until analysis. After a single freeze-thaw cycle, all serum samples were analyzed during the same session using an enzyme-linked immunosorbent assay (ELISA) with an ELISA kit (Human VEGF-A ELISA Kit; ELK Technology, Cat. No. ELK1129, Denver, USA) [12]. All samples were tested by the same clinician (BM) in the Department of Biochemistry to ensure consistency, and ophthalmic observers were masked to VEGF levels. 

At baseline, the laboratory tests for fasting and postprandial blood sugar, glycosylated hemoglobin (HbA1c), liver function tests, kidney function tests, and lipid profile were confirmed to be normal. For treatment of non-resolving CSCR, intravitreal injection of a ranibizumab biosimilar (RbB; Razumab; Intas, Ahmedabad, India) at a concentration of 0.3 mg/0.05 mL was administered under aseptic conditions. We used a 30-gauge needle to inject RbB at the superotemporal pars plana, approximately 3.5-4.0 mm behind the limbus. After withdrawal of the needle, a sterile cotton-tipped applicator soaked in 5% povidone-iodine was applied to the injection site to prevent reflux of the anti-VEGF. Postoperatively, 3% ciprofloxacin eye drops were prescribed four times daily for 10 days. Following anti-VEGF injection, IOP was assessed on day 1, while other ocular parameters were evaluated at one month. Patients were monitored for systemic complications related to anti-VEGF therapy, including arterial thromboembolic events, as well as ocular complications such as lens injury, glaucoma, retinal detachment, infection, and hemorrhage.

The serum VEGF-A levels and other ocular parameters were measured again at one month after treatment. A correlation was conducted between serum VEGF-A levels and ocular features, both at pre-treatment and after anti-VEGF injection. 

Sample size assessment

According to available literature (Hu et al.) [16], taking the percentage of patients showing a roundish dark halo around coarse granulated high reflective area on OCTA in CSCR patients as 81.8%, at 95% confidence level and with a relative error of 20%, the estimated sample size was 22, calculated using the appropriate statistical formula.

$n = \frac{Z_{\alpha}^{2} \, p \, q}{L^{2}}$

Where n=sample size; Zα=1.96 at 95% confidence level; p=percentage of patients showing roundish dark halo around coarse granulated high reflective area on OCTA in CSCR=81.8%; q=1-p=18.2%; L=relative error (20% of prevalence).

Therefore, the calculated minimum sample size was approximately 22 eyes. According to the central limit theorem, we needed a sample of at least 30 eyes. Hence, we included 31 eyes for this study.

Statistical analysis

All statistical analyses were performed using SPSS software version 25.0 (IBM Corp., Armonk, NY). The primary outcome parameters were FAZ and VD metrics on OCTA at baseline and one month after intravitreal RbB injection. The secondary outcome parameters included VA, CMT, SFCT, and serum VEGF-A levels before and after treatment, as well as the correlation of all ophthalmic parameters with serum VEGF-A levels at both time points. We presented the categorical variables as numbers and percentages. We assessed the normality of each variable using the Kolmogorov-Smirnov test. Quantitative data were expressed as mean and standard deviation or median (interquartile range), depending on the data distribution. At pre-treatment, VA, SFCT, FAZ perimeter and circularity, and ORCC foveal VD were parametric, while at post-treatment, SFCT, FAZ circularity, SCP parafoveal VD, DCP parafoveal VD, and DCP whole VD were parametric. The remaining data, including serum VEGF-A levels, were non-parametric. Hence, the correlation between serum VEGF-A levels and ophthalmic parameters was assessed using Spearman’s rho correlation coefficient. For controlling type 1 error in multiple comparisons, the Bonferroni correction was applied, and the adjusted significance threshold for the p-value was calculated.

## Results

In this study, we included 31 eyes from 31 CSCR patients, including 27 male (87.09%) and 4 female (12.90%) patients, with an average age of 39.23±6.9 years at presentation. The right eye was affected in 17 (54.8%) patients, while the left eye was involved in 14 (45.2%) patients. The average duration of CSCR disease was 457.13±533.31 days.

The mean fasting blood sugar was 98.49±13.63 (68-127) mg/dl, postprandial blood sugar was 121.94±12.11 (91-145) mg/dl, glycosylated hemoglobin (HbA1C) was 4.84±0.38 (4-5.8)%, blood urea was 25.11±5.8 (19-41) mg/dl, serum creatinine was 0.86±0.12 (0.6-1.2) mg/dl, serum glutamic-oxaloacetic transaminase (SGOT) was 30.83±12.73 (range: 16-75) units per litre and serum glutamic pyruvic transaminase (SGPT) was 39.45±16.16 (range: 12-84) units per litre.

All subjects presented with diminution of vision (31 eyes, 100%), while metamorphopsia was observed in 9 (29%) eyes and scotoma in 4 (12%) eyes. At the pre-treatment stage, the mean VA in LogMAR was 0.89±0.4 (0-1.77); the mean refractive error (RE) was -0.62±2.39 (-11-1.75), showing myopia; and BCVA in LogMAR was 0.81±0.43 (0-1.77). The mean IOP was 15.58±1.8 (12.2-18.5) mmHg, and the mean CS was 0.99±0.7 (0.05-1.85).

At the pre-treatment, the mean number of Ishihara plates 1-21 read was 15.77±8.43 (0-21) while the mean number of Ishihara plates 22-25 read was 2.97±1.7 (0-4). The Ishihara plates 22-25 were used to find red-green CV defects. On re-evaluation of the CV with the Farnsworth test, a normal CV was found in 3 (9.7%) eyes; mild tritanopia in 8 (25.8%), moderate tritanopia in 9 (29.0%), and severe tritanopia in 3 (9.7%) eyes. Protanopia was observed in 6 (19.3%) eyes and deuteranopia in 5 (16.1%) eyes. The apparent discrepancy in numbers is due to the presence of multiple types of CV defect in some eyes.

All affected eyes had central macular edema within 550 microns. The mean CMT was 286.81±117.67 (87-522) microns, and the mean SFCT was 194.32±62.76 (79-310) microns. Among 31 eyes, 24 (77.41%) eyes had NSD, and 25 (80.64%) eyes had PED. The NSD were located sub-foveally and parafoveally.

The FFA showed leakage at the fovea, appearing either as a well-defined, pinpoint leak or a small, round area of fluorescence, which later became pronounced and diffuse, creating a smokestack or inkblot-like pattern, centred around the macula. Some hyperfluorescent spots increased in intensity but not in size, indicating PED. Additionally, there were stippled fluorescence and window defects, indicating RPE changes/atrophy and areas of retinal damage due to prolonged fluid buildup.

The baseline blood sample analysis showed that the median serum VEGF-A level was 351.64 (interquartile range 243.57-478.71) pg/ml. This value was elevated compared to the normal range of 0 to 181 pg/ml [17], as reported in the published literature. Table 1 presents the correlation between pre-treatment serum VEGF-A levels and ocular and OCT parameters at baseline.

**Table 1:** Correlation of serum VEGF-A levels with ocular and optical coherence tomography features using Spearman correlation coefficient at the pre-treatment stage LogMAR: Logarithm of the Minimum Angle of Resolution

S. No.	Parameter	Correlation coefficient value	p value
1	Visual acuity (LogMAR)	0.118	0.527
2	Refractive error (Spherical equivalent)	-0.005	0.979
3	Best corrected visual acuity (LogMAR)	0.121	0.516
4	Intraocular pressure (mmHg)	-0.286	0.119
5	Contrast sensitivity	0.040	0.830
6	Number of Ishihara plates 1-21 read	-0.063	0.737
7	Number of Ishihara plates 22-25 read	-0.039	0.836
8	Central macular thickness (microns)	0.282	0.125
9	Sub-foveal choroidal thickness (microns)	0.006	0.977
10	Neurosensory detachment number	-0.077	0.680
11	Neurosensory detachment height (microns)	0.100	0.592
12	Neurosensory detachment width (microns)	0.019	0.919
13	Number of Pigment epithelial detachment	0.016	0.931
14	Pigment epithelial detachment height (microns)	0.246	0.181
15	Pigment epithelial detachment width (microns)	0.066	0.724

At the pre-treatment stage, serum VEGF-A correlated positively with the LogMAR values of VA and BCVA, CS, CMT, SFCT, NSD height and width, and PED number, height, and width. In contrast, it correlated negatively with the spherical equivalent of RE, IOP, the count of Ishihara plates correctly identified in both categories (i.e., 1-21 and 22-25), and NSD number. None of the correlation values was statistically significant.

The OCTA showed findings such as an enlarged and distorted FAZ, reduced vascularity, and projection artefacts from NSD in DCP, OR, and ORCC. Among OCTA parameters, the mean FAZ area was 0.25±0.18 (0.02-0.61) mm^2^, the FAZ perimeter was 2.49±1 (0.62-3.96) mm, and circularity was 0.44±0.14 (0.16-0.71). The mean VD in SCP whole was 33.06±14.26 (5-48), DCP whole 10.42±8.47 (0-27), OR whole 2.74±3.48 (0-13), ORCC whole 40.26±15.81 (2-56), CC whole 33.29±17.38 (0-53), and Ch whole was 36.74±15.92 (1-58).

Table 2 presents the correlation between pre-treatment serum VEGF-A levels and OCTA parameters at baseline.

**Table 2:** Correlation of serum VEGF-A levels with optical coherence tomography angiography features using Spearman correlation coefficient at pre-treatment stage FAZ: foveal avascular zone; SCP: superficial capillary plexus; VD: vessel density; DCP: deep capillary plexus; OR: outer retina; ORCC: outer retinal chorio-capillaries; CC: chorio-capillaries; Ch: choroid

S. No.	Parameter	Correlation coefficient value	p value
1	FAZ area (mm²)	-0.136	0.463
2	FAZ perimeter (mm)	-0.128	0.491
3	FAZ circularity	0.041	0.828
4	SCP foveal VD	0.306	0.094
5	SCP parafoveal VD	0.362	0.046
6	SCP perifoveal VD	0.223	0.226
7	SCP whole VD	0.320	0.080
8	DCP foveal VD	0.205	0.268
9	DCP parafoveal VD	-0.002	0.992
10	DCP perifoveal VD	0.070	0.706
11	DCP whole VD	0.020	0.914
12	OR foveal VD	0.093	0.616
13	OR parafoveal VD	0.000	0.999
14	OR perifoveal VD	-0.081	0.665
15	OR whole VD	-0.060	0.749
16	ORCC foveal VD	0.110	0.555
17	ORCC parafoveal VD	0.053	0.778
18	ORCC perifoveal VD	0.031	0.868
19	ORCC whole VD	0.055	0.767
20	CC foveal VD	0.041	0.826
21	CC parafoveal VD	0.098	0.600
22	CC perifoveal VD	0.188	0.310
23	CC whole VD	0.095	0.610
24	Ch foveal VD	-0.160	0.388
25	Ch parafoveal VD	-0.063	0.734
26	Ch perifoveal VD	0.092	0.622
27	Ch whole VD	-0.035	0.853

Before anti-VEGF treatment, serum VEGF-A levels showed a positive correlation with most OCTA features, strongest with SCP parafoveal VD (Rho=0.362; p=0.046). However, there was a negative correlation of the serum VEGF-A levels with FAZ area and perimeter, DCP parafoveal VD, OR perifoveal and whole VD, and Ch foveal, parafoveal and whole VD.

The analysis of blood samples taken one month after treatment showed that the median serum VEGF-A was 336.31 (interquartile range 259.455-500.105) pg/ml. This value was higher than the normal range of 0 to 181 pg/ml [17], as reported by earlier authors.

We found that the median difference between pre-treatment and post-treatment plasma VEGF-A levels was statistically not significant (p-value 0.891; Wilcoxon test applied). Notably, despite anti-VEGF treatment, the VEGF-A levels were elevated at one month after intravitreal RbB injection.

The anti-VEGF injection caused an improvement in mean VA to LogMAR 0.51±0.35 (0-1.5), mean BCVA to LogMAR 0.42±0.42 (0-1.5), and a reduction in the mean RE (myopia) to -0.62±2.05 (-10-1.5), i.e., eyes became less myopic. The mean IOP on day one was higher than the baseline value, and it was 20.45±5.5 (13-35) mmHg. The mean CS improved to 1.33±0.55 (0.05-1.85).

During examination on 1-21 Ishihara plates, it was found that the average count of plates 1-21 read at post-treatment increased to 17.23±7.78 (0-21) while the average count of Ishihara plates 22-25 read increased to 3.23±1.52 (0-4). We also examined the CV of study subjects with the Farnsworth test, and we found a normal CV in 4 (12.9%) eyes, mild tritanopia in 12 (38.7%), moderate tritanopia in 7 (22.6%), protanopia in 2 (6.4%), and deuteranopia in 6 (19.3%) eyes.

Following treatment, the mean CMT reduced to 256.65±109.57 (104-560) microns, and the mean SFCT increased to 209.84±41.53 (138-284) microns. Among 31 eyes, 17 (54.83%) eyes had NSD, and 24 (77.41%) eyes had PED.

The correlation between the post-treatment serum VEGF-A levels and ocular and OCT features at the post-treatment stage is shown in Table 3.

**Table 3:** Correlation of serum VEGF-A levels with ocular and optical coherence tomography features using Spearman correlation coefficient at post-treatment stage LogMAR: Logarithm of the Minimum Angle of Resolution

S. No.	Parameter	Correlation coefficient value	p value
1	Visual acuity (LogMAR)	0.263	0.153
2	Refractive error (Spherical equivalent)	-0.059	0.754
3	Best corrected visual acuity (LogMAR)	0.046	0.807
4	Intraocular pressure (mmHg)	-0.011	0.954
5	Contrast sensitivity	-0.143	0.443
6	Number of Ishihara plates 1-21 read	-0.274	0.136
7	Number of Ishihara plates 22-25 read	-0.352	0.053
8	Central macular thickness (microns)	0.063	0.736
9	Sub-foveal choroidal thickness (microns)	0.094	0.613
10	Neurosensory detachment number	0.290	0.114
11	Neurosensory detachment height (microns)	0.269	0.143
12	Neurosensory detachment width (microns)	0.289	0.116
13	Pigment epithelial detachment number	0.306	0.094
14	Pigment epithelial detachment height (microns)	0.204	0.269
15	Pigment epithelial detachment width (microns)	0.124	0.506

At the post-treatment stage, serum VEGF-A showed a positive correlation with the LogMAR values of VA and BCVA, CMT, SFCT, NSD number, height, and width, and with PED number, height, and width. A negative correlation was observed with the spherical equivalent of RE, IOP, CS, and the frequency of correctly read Ishihara plates in both categories, i.e., 1-21 and 22-25. None of the correlation values was statistically significant.

The OCTA showed a reduction in FAZ size, increased vascularity of retino-choroidal layers, and a projection artefact of NSD in DCP, OR, and ORCC. Among OCTA parameters, the mean FAZ area was 0.3±0.23 (0.02-1.16) mm^2^, the FAZ perimeter was 2.71±1.16 (0.68-6.75) mm, and circularity was 0.44±0.13 (0.17-0.67). The mean VD increased in different layers and in SCP whole was 35.58±10.91 (0-49), DCP whole 12.81±9.3 (0-41), OR whole 3.68±4.93 (0-17), ORCC whole 45.61±11.51 (6-57), CC whole 38±14.8 (5-54), and Ch whole was 41.71±11.94 (10-54).

Table 4 shows the correlation between post-treatment serum VEGF-A levels and OCTA features at the post-treatment stage.

**Table 4:** Correlation of serum VEGF-A levels with optical coherence tomography angiography features using Spearman correlation coefficient at post-treatment stage FAZ: foveal avascular zone; SCP: superficial capillary plexus; VD: vessel density; DCP: deep capillary plexus; OR: outer retina; ORCC: outer retinal chorio-capillaries; CC: chorio-capillaries; Ch: choroid

S. No.	Parameter	Correlation coefficient value	p value
1	FAZ area (mm²)	-0.156	0.401
2	FAZ perimeter (mm)	-0.114	0.540
3	FAZ circularity	-0.335	0.066
4	SCP foveal VD	-0.214	0.248
5	SCP parafoveal VD	-0.208	0.260
6	SCP perifoveal VD	-0.270	0.142
7	SCP whole VD	-0.282	0.124
8	DCP foveal VD	-0.326	0.074
9	DCP parafoveal VD	-0.246	0.182
10	DCP perifoveal VD	-0.139	0.455
11	DCP whole VD	-0.225	0.224
12	OR foveal VD	0.188	0.310
13	OR parafoveal VD	0.130	0.483
14	OR perifoveal VD	0.056	0.766
15	OR whole VD	0.040	0.830
16	ORCC foveal VD	-0.060	0.749
17	ORCC parafoveal VD	-0.181	0.327
18	ORCC perifoveal VD	-0.318	0.081
19	ORCC whole VD	-0.295	0.107
20	CC foveal VD	-0.192	0.299
21	CC parafoveal VD	-0.227	0.219
22	CC perifoveal VD	-0.408	0.023
23	CC whole VD	-0.312	0.087
24	Ch foveal VD	-0.287	0.117
25	Ch parafoveal VD	-0.254	0.168
26	Ch perifoveal VD	-0.338	0.063
27	Ch whole VD	-0.274	0.135

After treatment, all FAZ indices and VD in all layers (across the foveal, parafoveal, perifoveal, and whole regions), except OR, demonstrated a negative correlation with serum VEGF-A levels. However, none of the correlations reached statistical significance.

Because the Rho correlation coefficients were very low, the correlations computed in this study were weak. To account for multiple comparisons, the Bonferroni correction was applied to control for type I error. The adjusted significance thresholds were p<0.003 for ocular and OCT parameters (15 correlations; Tables 1 and 3) and p<0.002 for OCTA parameters (27 correlations; Tables 2 and 4). Thus, none of the correlations was statistically significant. The subjects did not experience any ocular or systemic side effects from intravitreal RbB.

## Discussion

The exact pathogenesis of CSCR remains complex and multifactorial, but a significant contributor appears to be dysfunction of the RPE, leading to leakage of fluid from choroidal vessels into the subretinal space [2]. Anti-VEGF agents, such as ranibizumab, aflibercept, and bevacizumab, work by inhibiting VEGF, a critical mediator of abnormal vascular leakage and retinal edema [5].

Very few authors have studied the role of anti-VEGF therapy in CSCR, and these studies have generally focused on VA and OCT features. Zhao and Zhang studied 12 patients with a mean age of 49.4±7.7 years and used three anti-VEGF agents: aflibercept in eight eyes, ranibizumab in four eyes, and conbercept in one eye. These patients had either visible CNVM or putative CNVM. At one week post-injection, CMT and SRF were significantly reduced from 420.6±128.6 to 237.2±42.2 microns and from 209.4±129.0 to 55.2±38.3 microns, respectively [7].

Ji et al. conducted a meta-analysis of 14 studies involving 266 CSCR eyes and found that anti-VEGF treatment with aflibercept, ranibizumab, or bevacizumab was not superior to observation at a six-month follow-up for acute CSCR. In patients with chronic CSCR, anti-VEGF agents resulted in significant improvement in both CMT and BCVA. The meta-analysis suggested that anti-VEGF medications may be a viable option for the treatment of chronic CSCR [2].

Park et al. conducted a comparative study of 21 eyes with acute CSCR and 15 eyes in the observation group. At baseline, the two groups did not differ significantly in BCVA or CMT. On examination at 1, 3, 6, 9, and 12 months, BCVA and CMT improved significantly in both groups. The time to complete the resolution of SRF was significantly shorter in subjects who received anti-VEGF therapy. Additionally, eyes in the anti-VEGF group had a significantly higher rate of the IS/OS line preservation (p=0.023) [4].

Only a limited number of studies have investigated OCTA features in CSCR. Zhang et al. conducted a retrospective study in 16 eyes of 15 patients with a mean age of 51.1±8.0 years and an average follow-up of 3.90±3.37 months. All affected eyes had SRF and PED, while six had a well-defined CNV and 10 had putative CNV. The patients were treated with anti-VEGF agents, including ranibizumab, conbercept, and aflibercept. Following treatment, the VA improved significantly from LogMAR 0.189±0.145 to 0.087±0.081 (p=0.0097), CMT reduced from 381.77±84.10 μm to 225.09±41.17 μm (p<0.0001), and the height of SRF reduced from 204.77±86.90 μm to 38.25±50.01 μm (p<0.0001). The OCTA showed that while the visible CNV area remained virtually unchanged, the area of putative CNV displayed an obvious reduction. The average number of hyperreflective foci also reduced significantly from 5.56±4.95 to 2.69±3.35 in the OR and CC layer [5].

Despite studying the effects of anti-VEGF agents, the authors did not measure VEGF-A levels in their subjects or examine the correlation between VEGF-A levels and ocular features. However, these studies strengthen the relevance of studying VEGF-A levels in CSCR and further inspire us to examine the relationship between VEGF-A levels and ocular features, especially OCTA parameters.

Few authors have examined inflammatory factors, including VEGF, in CSCR. In one such study, Mao et al. measured inflammatory factors, including VEGF, monocyte chemoattractant protein-1 (MCP-1), IL-6, IL-8, and IL-10 in the aqueous humour of 88 chronic CSCR patients and 30 controls. The authors divided patients into three groups: flat irregular PED (FIPED) with CNVM, FIPED without CNVM and focal PED. They found that VEGF was the only cytokine significantly elevated in patients with FIPED regardless of CNVM presence. One month after intravitreal conbercept (0.5 mg/0.05 ml), it was seen that the intervention was significantly more effective in FIPED with or without CNV than in focal PED (p<0.05). Both the FIPED groups showed statistically significant improvement in BCVA, though the focal PED group did not. All three groups showed significant decreases in CMT (p<0.05), but only FIPED patients showed a significant reduction in PED height (p<0.05). In the FIPED group, the aqueous humour VEGF level was 48.33±16.51 pg/ml in subjects with CNV, which was significantly higher than the VEGF level of 42.72±14.39 pg/ml observed in subjects without CNV (p=0.234). The VEGF levels were 34.02±11.12 and 29.66±13.24 pg/ml in the focal PED and control groups, respectively, and signiﬁcantly lower than the VEGF levels in FIPED with or without CNV. After intravitreal conbercept injection, VEGF levels decreased significantly at the one-month follow-up visit. Conversely, IL-6 levels increased (p=0.016). The levels of other cytokines and MCP-1 did not alter significantly [18]. Mao et al. concluded that the presence of FIPED is an indication for anti-VEGF treatment, while it was not so for focal PED, which would not benefit much. Thus, OCT features can guide treatment strategy [18].

Given the paucity of studies on VEGF-A levels and their correlation with OCT and OCTA features, the role of VEGF-A in the pathogenesis, manifestations, and clinical course of CSCR remains incompletely elucidated. Our investigation found that VEGF-A levels correlated weakly with the OCTA features. Thus, it is unclear whether OCTA features can guide treatment strategy, i.e., whether anti-VEGF therapy will be beneficial. Our study suggests that anti-VEGF agents will reduce CMT, SFCT, NSD, and PED, but these will increase FAZ area and perimeter. Higher VEGF levels were associated with reduced FAZ area and perimeter, likely because VEGF, as an inflammatory factor, increases vascularity. However, in the case of VD, contradictory results were seen. Higher VEGF-A levels were generally accompanied by increased VD in all layers except OR and Ch at pre-treatment. However, an increase in VEGF-A level was observed with a reduced VD in all layers except OR at post-treatment. Hence, it appears that the effect of anti-VEGF agents on VD cannot be foretold. 

A previous correlation study in subjects with diabetic macular edema (DME) showed similar findings. Higher VEGF-A levels accompanied decreased visual functions, including VA, BCVA, CS, and CV, as well as lower IOP. As in the present CSCR study, both the FAZ area and perimeter in DME showed negative correlations with serum VEGF-A levels. FAZ circularity showed a positive correlation before RbB injection but a negative correlation after RbB injection [12].

This study has some limitations. First, the sample size was relatively small, which may affect the generalisability of the findings. Second, the study lacked a control group for comparison. The OCTA artefacts secondary to the pressure effects of NSD or PED may have influenced VD measurements. Finally, the observation and follow-up period was short. Therefore, large, controlled, prospective, and multi-centre studies are needed to clarify the impact of VEGF-A on ocular, OCT, and OCTA features of CSCR. Then it would be possible to assess and thereby utilise intravitreal anti-VEGF agents more effectively for the treatment of CSCR. Baharivand et al. reported a significant correlation between vitreous and serum VEGF levels [19]. However, it is likely that changes in other inflammatory factors, including cytokines, may have acted as systemic confounders and influenced the results of the present study. Therefore, we believe that serum VEGF-A levels may not accurately reflect intraocular VEGF-A concentrations. The presence of such systemic confounding factors may also explain the elevated serum VEGF-A levels observed following treatment. At this juncture, we also recommend similar studies using newer anti-VEGF drugs. The authors intend to publish additional and distinct findings derived from the same research in subsequent publications.

## Conclusions

This study showed the extent to which VEGF-A levels affected OCTA features in CSCR and how the anti-VEGF agent influenced this relation. Higher serum VEGF-A was associated with worse VA and increased CMT, SFCT, NSD height and width, and PED number, height, and width. Elevated VEGF-A levels coexisted with lower IOP and RE values and a worse CV on Ishihara plates. During OCTA, a rise in VEGF-A was associated with a reduction in FAZ area and perimeter. At baseline, higher levels of VEGF-A were accompanied by an increase in VD in all layers except OR and Ch. At one month following intravitreal RbB injection, serum VEGF-A levels were higher than pre-treatment levels. Regarding correlation, FAZ parameters showed a pattern similar to that observed in the pre-treatment stage. However, increased VEGF-A was found to be coupled with decreased VD in all retino-choroidal layers except OR. As VD in Ch showed a persistent negative correlation with VEGF-A levels, this study does not indicate that increased VEGF-A levels are responsible for causing choroidal hypervascularity and thereby resultant leakage in CSCR. This study is a preliminary investigation aimed at exploring the correlation between OCTA parameters and serum VEGF-A levels. The authors emphasize that all observed correlations were weak and not statistically significant; furthermore, correlation does not necessarily imply causation. Thus, the results need to be viewed carefully. 
